# Phylogenetic inference under varying proportions of indel-induced alignment gaps

**DOI:** 10.1186/1471-2148-9-211

**Published:** 2009-08-23

**Authors:** Bhakti Dwivedi, Sudhindra R Gadagkar

**Affiliations:** 1 Department of Biology, University of Dayton, 300 College Park, Dayton, OH 46469-2320, USA; 2 Department of Natural Sciences, PO Box 1004, 1400 Brush Row Rd, Wilberforce, Ohio 45384, USA

## Abstract

**Background:**

The effect of alignment gaps on phylogenetic accuracy has been the subject of numerous studies. In this study, we investigated the relationship between the total number of gapped sites and phylogenetic accuracy, when the gaps were introduced (by means of computer simulation) to reflect indel (insertion/deletion) events during the evolution of DNA sequences. The resulting (true) alignments were subjected to commonly used gap treatment and phylogenetic inference methods.

**Results:**

(1) In general, there was a strong – almost deterministic – relationship between the amount of gap in the data and the level of phylogenetic accuracy when the alignments were very "gappy", (2) gaps resulting from deletions (as opposed to insertions) contributed more to the inaccuracy of phylogenetic inference, (3) the probabilistic methods (Bayesian, PhyML & "ML*ε*, " a method implemented in DNAML in PHYLIP) performed better at most levels of gap percentage when compared to parsimony (MP) and distance (NJ) methods, with Bayesian analysis being clearly the best, (4) methods that treat gapped sites as missing data yielded less accurate trees when compared to those that attribute phylogenetic signal to the gapped sites (by coding them as binary character data – presence/absence, or as in the ML*ε *method), and (5) in general, the accuracy of phylogenetic inference depended upon the amount of available data when the gaps resulted from mainly deletion events, and the amount of missing data when insertion events were equally likely to have caused the alignment gaps.

**Conclusion:**

When gaps in an alignment are a consequence of indel events in the evolution of the sequences, the accuracy of phylogenetic analysis is likely to improve if: (1) alignment gaps are categorized as arising from insertion events or deletion events and then treated separately in the analysis, (2) the evolutionary signal provided by indels is harnessed in the phylogenetic analysis, and (3) methods that utilize the phylogenetic signal in indels are developed for distance methods too. When the true homology is known and the amount of gaps is 20 percent of the alignment length or less, the methods used in this study are likely to yield trees with 90–100 percent accuracy.

## Background

DNA sequences are used routinely to infer phylogenies [1-3]. The sequences within lineages (branches of the phylogenetic tree) evolve independently over time by means of several evolutionary processes, including point replacements of nucleotides (base substitutions), and insertion and deletion (indel) events. While base substitutions change the nucleotide composition of a given sequence, indels are likely to change the total length of the sequence. If indel events have occurred during the course of evolution of the molecular sequences being studied, it becomes necessary to align the corresponding homologous regions among the sequences for a proper site-by-site comparison among them, before phylogenetic analysis. In the process of alignment, gaps are introduced in the sequences to account for the indels. Different methods have been devised for dealing with gapped sites during phylogenetic analysis, ranging from ignoring the gapped sites from the alignment to inferring or differentially coding the state at each gapped site, using a number of different methods (for a list of methods, see [4-6]). Most of these treatment methods work reasonably well when the proportion of gapped sites in an alignment is small [5,6].

There are many examples in the literature of studies that have used molecular sequences (DNA and protein) with rather large gaps to infer phylogenies [7-9]. It appears logical to expect an inverse relationship between the proportion of gapped sites in an alignment and the accuracy of the inferred phylogeny, particularly if the gaps are not treated as reflective of distinct evolutionary events, and thus, containing distinct phylogenetic signal. However, the relationship between the extent of "gappiness" in the data resulting from indel events in the evolutionary history of the sequences on the one hand, and phylogenetic accuracy on the other, has not been studied by introducing and systematically varying the number of gaps in the alignments in a biologically realistic manner, even as the literature on alignment gaps in the phylogenetic context has increased of late [6,10-15]. For example, several studies investigating the relationship between the amount of alignment gap and phylogenetic accuracy have done so in the context of aligning sequence fragments such as ESTs (e.g., [12,13]), using computer simulation to first generate the alignments and then introduce gaps, such that the gaps do not contain any phylogenetic signal (e.g., [10,11]); are in the context of only empirical data (e.g., [8]); or where the emphasis was more on levels of divergence among the taxa (e.g., [16]). Furthermore, the relative performance of the gap treatment methods that are common among inference methods has also not been compared in this context. For example, all inference methods allow gaps to be treated as missing data or "MD" (although the treatment of the missing data differs among the methods, with the state at the gapped sites inferred in parsimony and distance-based methods of phylogenetic analysis, based on criteria that are specific to each method, while in likelihood and Bayesian analyses, the likelihoods are summed over all four possible assignments of a nucleotide to a given gapped site). It is not known how the data inferred under these criteria work in conjunction with each of the respective inference methods to influence the accuracy of phylogenetic inference, when the gaps reflect indel events in the alignment.

**Figure 1:**
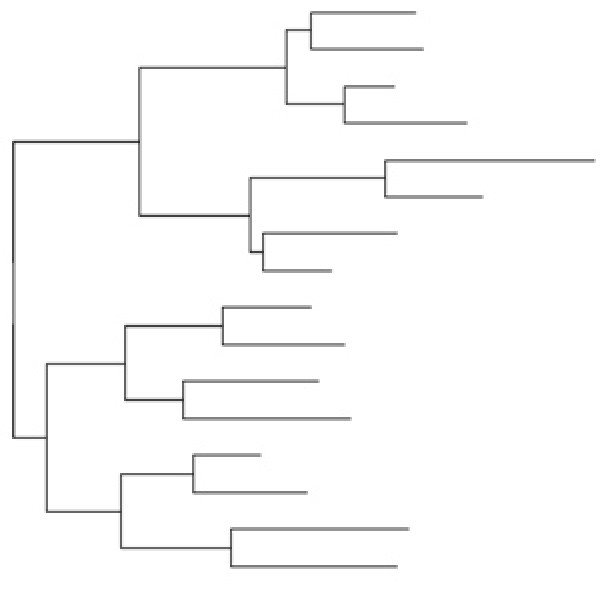
**The model tree**. The 16-taxon balanced tree, obtained from Ogden and Rosenberg (2006), was used as a model tree for the simulations of DNA evolution, the results of which we have presented in this paper. Two more sets of simulations were also done – one with a random-branching tree and the other with a pectinate tree (both 16-taxon too), but the results from these analyses have not been shown, for reasons explained in the text. The scale given in the figure refers to the number of nucleotide substitutions per site. During simulation, the total number of substitutions to be made for a given branch, for a given parameter-combination, was obtained as the product of the branch length in the model tree, the rate multiplier and the sequence length. Values from the latter two parameters were obtained from Additional file 1.

We obtained sequence alignments for this study by means of simulating non-coding DNA sequence evolution, introducing nucleotide point substitutions (replacements) and insertion/deletion (indel) events along a balanced (symmetrical) 16-taxon model tree (Figure 1). (Simulations were done along random and pectinate 16-taxa trees as well, but we report the results only from the balanced model tree shown in Figure 1 for reasons explained below.) The simulations were done while systematically varying the values of different sequence and indel parameters. All the simulation parameters were varied to include biologically realistic values. For example, the rate of introduction of indels included the range seen in non-coding sequences [17-20]. Similarly, the ratio of insertion to deletion events was also varied based on published results [18,20-22]. It was important to vary the ratio of insertions to deletions in order to determine if there was a differential effect on phylogenetic accuracy, since most of the commonly used gap treatment methods do not differentiate between gaps resulting from the two types of evolutionary events.

We assessed the accuracy of phylogenetic inference as the topological correctness of the inferred tree when compared to the model tree. Our results show that overall, when the percentage of gapped sites (cells in the alignment matrix) in the alignment is low (≤ 20 percent), all the inference methods (using any gap-treatment method) perform well (with 90–100% accuracy). On the other hand, when the number of gapped sites increases in the alignment, the probabilistic methods (particularly Bayesian analysis) are clearly more accurate, although at the highest gap levels, NJ and MP are sometimes better. Our results also show that gaps resulting from deletion events in the evolutionary history of the sequences appear to be harder to reconcile (when compared to those resulting from insertion events), leading to greater inaccuracies in phylogenetic inference, evidently because of the loss of the phylogenetic signal present in the sites deleted. When compared to MD method of treating gaps, a much higher accuracy was seen in our study when the gaps were coded separately as in the BC (Binary Character state) treatment in conjunction with the Bayesian and MP methods, or as in the DNAML*ε *package [23].

## Results

We first describe the manner in which the alignment gaps were quantified in this study, and the effect of different simulation parameters on the number of gaps in the alignment.

### Quantification of alignment gaps

The amount of gap in an alignment was determined as a percentage, in the following manner. First, the number of gapped sites was determined for each sequence and then obtained as an average among all the sequences in the alignment. (Note that our definition of a gapped site is common to all methods: a *single *cell in the alignment matrix. Thus, a gap that covers three cells (the space of three bases) in a given sequence, even if contiguous, is counted as three gapped sites in that sequence.) This was expressed as a percentage of the altered length (as a result of indel introduction) of the alignment. This percentage was then averaged across the replicates, for a given treatment, as a simple arithmetic mean (since the change in length due to the introduction of the indels varied minimally among them). We refer to the gap percentage by the term G/S (for Gap percentage per Sequence) throughout the paper. The gap percentages thus obtained (G/S) were used to compare the relative performances of the phylogenetic methods (PhyML, MP, NJ, and Bayesian analysis) under the different gap treatment methods (MD, BC, and ML*ε*).

**Figure 2:**
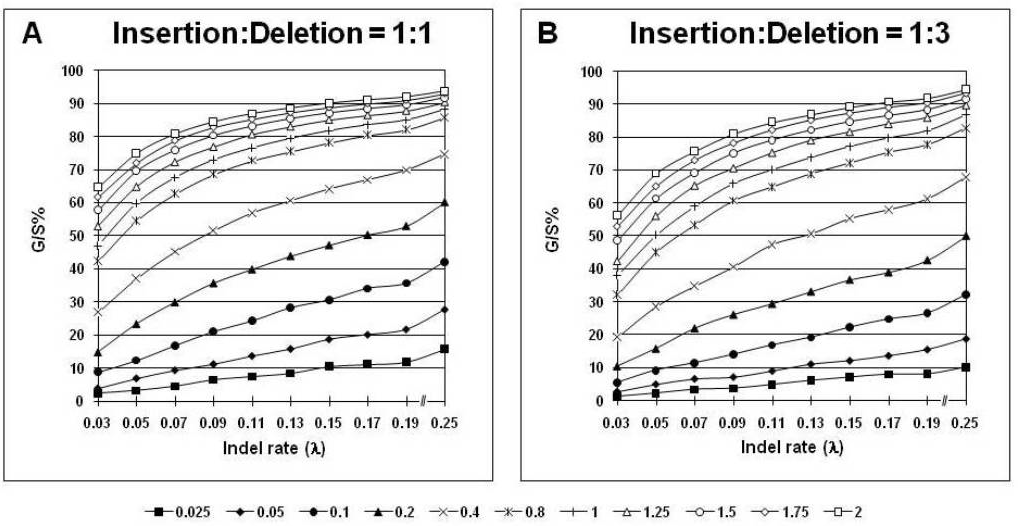
**Distribution of alignment gap percentages**. The percentage of alignment gaps, G/S, given as the number of gapped sites per sequence length averaged over all the sequences in the alignment and expressed as a percentage of the alignment length altered after indel introduction, is plotted as a function of the indel rate (*λ*) for each nucleotide substitution rate, *r*, whose values are shown by means of different markers; see legend below figure. G/S is a suitable quantification of gaps when they are treated as missing data, binary characters or in the ML*ε *method. G/S values were averaged over all the other parameter values for sequence length, *l *= 500, gamma distribution shape parameter, *α *= 0.5, and transition-transversion rate ratio, *κ *= 2. The gap distributions are shown for insertion-deletion rate ratios of 1:1 (Panel A), and 1:3 (Panel B).

Figure 2 shows the G/S distribution of the total number of gaps in an alignment, expressed as a percentage of the total length of the alignment. Panels A and B refer to simulations where the rate ratio of insertions to deletions was 1:1 and 1:3, respectively. In each panel, the distribution of gaps has been plotted separately for each substitution rate (*r*), as a function of the rate of indel introduction (*λ*) that in turn, was varied as a function of the substitution rate. Both panels of Figure 2 show that the average gap percentage increases nonlinearly with increase in *λ *for all *r*. The gap percentage was minimum (~2%) when *λ *= 0.03, and *r *= 0.025, and maximum (~90%) when *λ *≥ 0.19 and *r *≥ 1.0. No noticeable differences were seen in the percentage of gaps in the alignments when different values of sequence length (*l*) and transition-transversion rate ratio (*κ*) were used in the simulations (not shown). However, as expected, the gap percentage varied considerably with the relative proportion of insertions and deletions, with more gaps seen when the ratio of insertions to deletions was 1:1 and fewer when the ratio was 1:3, especially at low to medium substitution rates. This difference in the number of gaps is because an insertion event in a single sequence adds a gap of the size of the insertion to all the other sequences during alignment, whereas a deletion in a sequence results in a gap in only that sequence and no other, especially if the insertion/deletion event is recent in the evolutionary history of the affected sequence. The distribution of gap percentages for the random and pectinate trees was largely similar to that shown for the balanced tree in Figure 2.

### Finding the gap threshold

**Figure 3:**
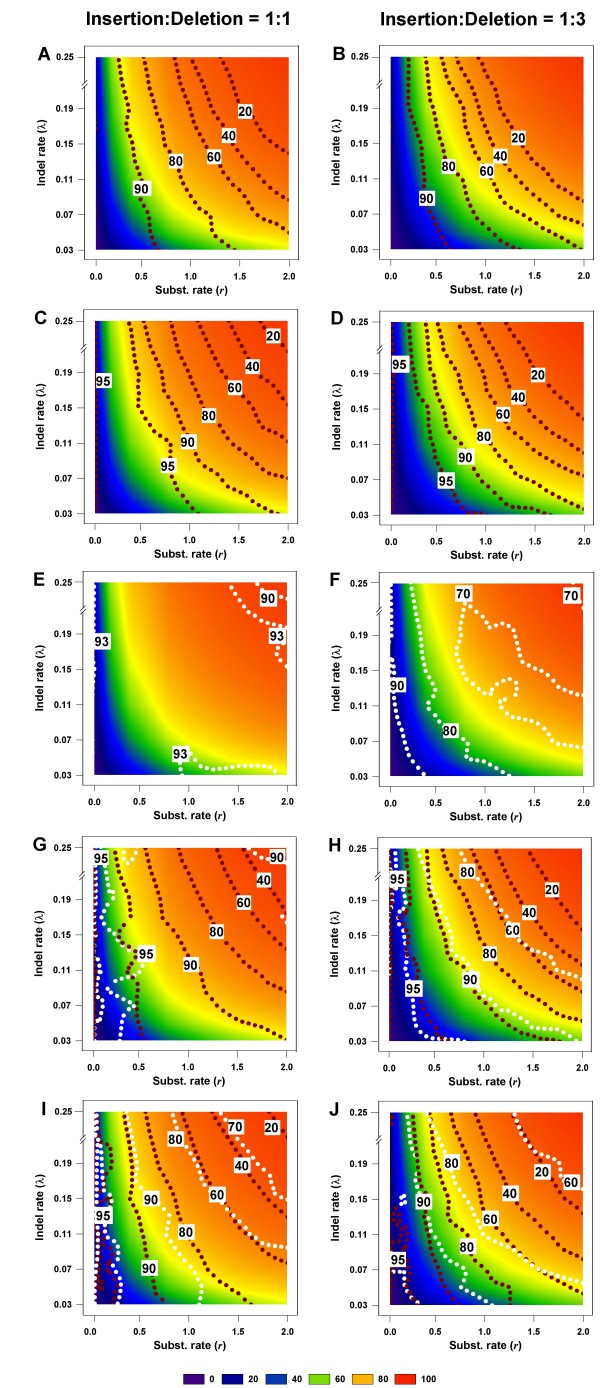
**Gap thresholds for different levels of phylogenetic accuracy**. The gap thresholds are shown for NJ (Panels A, B), PhyML (C, D), ML*ε *(E, F), Bayesian (G, H), and MP (I, J) methods, for various levels of phylogenetic accuracy. The background in each graph is color-coded to reflect the gap percentage thresholds; see legend at bottom of figure. Each graph also shows values of  plotted against different gap thresholds. In Panels E-F, and G-J, white dotted lines refers to the ML*ε *method of Rivas and Eddy (2008), and the Binary Character state (BC) treatment, respectively. The dark red dotted lines refer to the Missing Data (MD) method in all panels. Each  value reflects one of all possible combinations of values of substitution rate, *r *and indel rate, *λ*, (see Additional file 1), sequence length, *l *= 500, transition-transversion rate ratio, *κ *= 2, and the gamma among-site rate variation shape parameter, *α *= 0.5, averaged over 100 replicates, for a total of 110 data points in each graph. The left panels show the results for the insertion-deletion rate ratio of 1:1 and the right panels for the ratio 1:3.

Using the above measures of phylogenetic accuracy, it is possible to determine thresholds of gap percentages for given levels of phylogenetic accuracy. These thresholds are shown in Figure 3, which is arranged such that there are two panels for each inference method, one for the insertion-deletion rate ratio of 1:1 and the other for the ratio 1:3. The horizontal and vertical axes in each panel reflect the rate of nucleotide substitution and rate of indel introduction, respectively. However, in order to relate to empirical phylogenetic analyses (where these rates are not routinely determined), the background in this figure has been color-coded based on the percentage of alignment gaps (which can be easily determined), and the contour lines of phylogenetic accuracy have been drawn against this background. Thus, one can trace the level of accuracy of phylogenetic reconstruction based on the percentage of gaps in the alignment rather than on the rates of substitution or indel introduction. Such a representation also makes it easier to determine gap thresholds for phylogenetic accuracy in empirical studies, to determine the expected level of accuracy given a certain percentage of gaps in an alignment.

In Figure 3, Panels A and B show the level of phylogenetic accuracy for gap treatments in the NJ analysis. These results are remarkable for several reasons. First, the contour lines of accuracy typically follow specific gap percentage ranges, as indicated by the color of the background. In other words, there appears to be a somewhat deterministic relationship between the number of gaps in an alignment and the level of phylogenetic accuracy one can expect in an NJ analysis. This appears to be true in the case of PhyML also (Panels C and D). Furthermore, both methods can be seen to be doing better in the 1:1 than in the 1:3 panels, showing that the relative proportions of insertions and deletions matter in determining the accuracy.

Panels E and F show the results for ML*ε *analysis. Here, we see that the minimum accuracy is approximately 90% and 70% for the 1:1 and 1:3 cases, respectively. Clearly, the ML*ε *analysis has higher accuracy when compared to the MD analysis in conjunction with any inference method. The integrated method incorporating both substitutions and indels, ML*ε *appears to be equivalent in accuracy to the BC method in Bayesian analysis, in the case of the 1:1 ratio of insertions and deletions. However, the accuracy of ML*ε *is lower for datasets with larger deletion biases (as in the 1:3 ratio) and is in keeping with the other indel-coding methods (Panels G-J).

The Bayesian and MP analyses are shown in the panels, G, H, and I, J, respectively, with the dark red and white dotted contour lines within each panel representing the accuracy when the gaps are treated as Missing Data (MD) and Binary Characters (BC), respectively. As in the case of the other methods, the relationship between accuracy and G/S is clearly strong here too. Furthermore, this apparent cause-and-effect relationship appears to hold, whether the treatment method is MD or BC, especially at larger G/S values (towards the red end of the background color). It must, however, be noted that the actual relationship between the percentage of gaps in an alignment and the level of phylogenetic accuracy that can be expected is vastly different between the two gap treatment methods, MD and BC. Thus, even when the G/S value exceeds 80 percent of the length of the alignment (orange color background), as much as 70 percent of the branches are reconstructed accurately by MP (Panel I), and 90% by the Bayesian method (Panel G), when the gaps are treated as binary characters (BC), and the insertion-deletion ratio is 1:1. In contrast, only approximately 40 percent of the branches are accurately inferred in either analysis under the MD treatment (same panels). The only case where the relationship between the percentage of gaps and phylogenetic accuracy is not as straightforward is at very high accuracy levels; the contour lines for 95 percent accuracy cross color (gap percentage) boundaries or are confined to small portions of the gap percentage range of 0–20 percent. The reconstruction accuracy for the Bayesian and MP methods in alignments where the gaps are largely due to deletion events (1:3) is worse than when compared to alignments where there is equal contribution from insertions and deletions to the gaps (1:1). Panels G and H (Bayesian analysis) and I and J (MP) show that there is a 10–20 percent difference in accuracy for a given level of gaps in an alignment between the two insertion-deletion ratios, whether for MD or BC treatments. Thus, when the gaps exceed 90 percent of the alignment length, the reconstruction accuracy is seen to be around 90 percent (BC treatment) and approximately 40 percent (MD treatment) when the insertion-deletion ratio is 1:1, whereas it is less than 80 percent (but more than 60 percent; BC treatment) and 20 percent (MD treatment) when the ratio is 1:3 (Panels G and H).

Increasing the sequence length does not appear to change the pattern of these results very much, except that there is greater accuracy when the sequence length is 2500 nts. (not shown). This improvement in accuracy, however, is not uniform across the breadth of the gap percentage landscape, being higher at the low gap percentage levels. For example, when the sequence length is 500 nts., and the gaps are equal to or greater than 80 percent, the accuracy is approximately 80 percent (Figure 3, Panel I; BC treatment). The corresponding accuracy when the sequence length is 2500 nts., is approximately 99 percent – a 9–10% difference between the two lengths. Sequence length is known to be an important determinant of phylogenetic accuracy [24-26] and its influence is not being investigated in this study.

Figure 3 also shows that there are some differences among the inference methods with respect to the gap threshold. First, the level of accuracy at a given gap percentage is higher in the Bayesian, PhyML and MP analyses when compared to the other analyses, especially at higher gap percentages, when the comparison is made for the MD treatment (which is common among the inference methods, except of course, ML*ε*, which is an integrated gap-coding/phylogenetic analysis method). Thus, when the gaps amount to more than 80 percent in the alignment (for insertion-deletion rate ratio of 1:1), the Bayesian, MP, and PhyML-analyzed trees are inferred with approximately 60%, 40%, and 40% accuracy, respectively, whereas the accuracy in the NJ analyses is less than 20 percent. The comparison here also reflects the differences among the criteria used in treating the gaps as missing data in the three inference methods. From these results, it appears that the MD treatment in NJ infers the states at the gapped sites less accurately than does the corresponding treatment in MP and the method used in assigning the likelihood in Bayesian and PhyML analyses. Furthermore, the tightness of the relationship between the contour lines of phylogenetic accuracy and the gap percentages on the one hand, and the lower accuracy at high gap percentages in the PhyML and NJ analyses on the other, imply that while these two methods appear to be more capable of overcoming other sources of error in phylogenetic inference (such as homoplasy in the case of MP), they fall victim to poorer treatment of gaps as missing data.

### Phylogenetic accuracy of different inference methods under varying gap percentages

**Figure 4:**
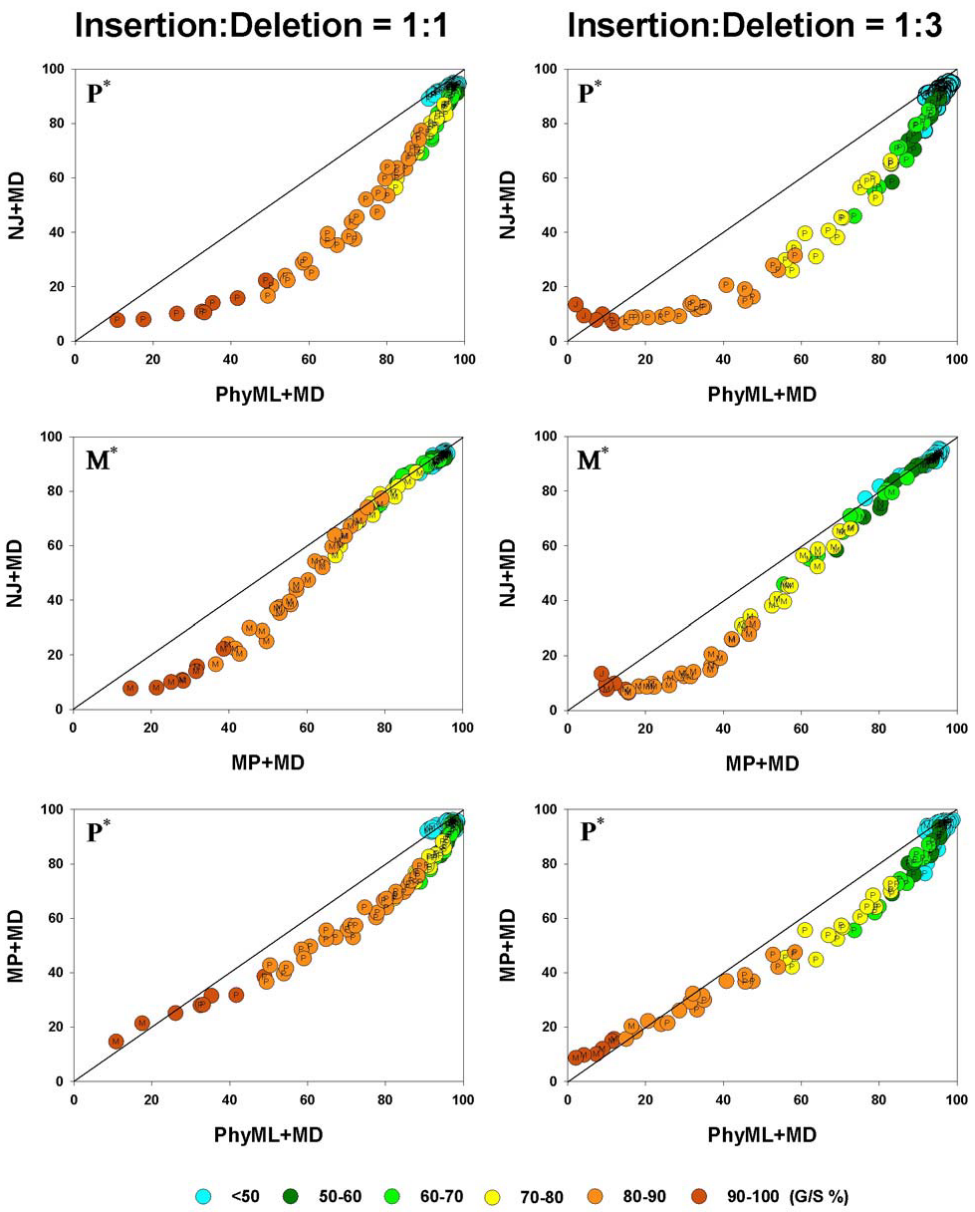
**Pairwise comparison of inference methods under MD gap treatment**.  values are compared, in a pairwise fashion, for four inference methods: NJ, PhyML, and MP. As elsewhere, the left and right columns refer to the 1:1 and 1:3 insertion-deletion rate ratios. In each panel, the average phylogenetic accuracy, , for one inference method is plotted against that of another. The dots in each graph are color coded to reflect the gap percentage (G/S %) against which the  values have been measured, ranging from light blue (for the lowest G/S values) to red (for the highest G/S values); see legend below figure. Each  value reflects one of all possible combinations of values of substitution rate, *r *and indel rate, *λ*, (see Additional file 1), sequence length, *l *= 500, transition-transversion rate ratio, *κ *= 2, and the gamma among-site rate variation shape parameter, *α *= 0.5, averaged over 100 replicates, for a total of 110 data points in each graph. The paired *t*-test (*p *< 0.05) results are shown with a letter (within the dot) that signifies if a particular method is statistically better than the other in a given comparison (J – Neighbor-Joining, P – PhyML, and M – Maximum Parsimony) for the parameter combination. The *t*-test results that were not statistically significant are presented with no symbol (letter) within each dot. The results of the Z test (*p *< 0.001) over 110 data points for each method-method comparison is shown with a letter followed by an asterisk (J* – Neighbor-Joining, P* – PhyML, and M* – Maximum Parsimony)

We first compare the phylogenetic accuracy of all the inference methods, taken two at a time, for the MD gap treatment since this is available for all the methods. The results are shown in Figure 4. In each panel in Figure 4, the average phylogenetic accuracy, , for one method is plotted against that of another, so that if identical, the two  values will lie on the diagonal. Values above the diagonal refer to cases where the method plotted on the vertical axis has relatively higher  values and those below the diagonal to the cases where the method on the horizontal axis has the higher  values.

For each comparison between inference methods, the left panel shows the results for the insertion-deletion ratio 1:1, and the right panel for the ratio 1:3. In each panel, the graph is also color-coded to reflect the gap percentage (G/S) against which the  values have been measured, with the color ranging from light blue (for the lowest G/S values) to red (for the highest G/S values). In general all the inference methods do rather poorly when G/S is extremely high and very well when G/S is very low. Furthermore, as seen in Figure 3, all methods yield more accurate trees for a given G/S value when the gaps are caused by insertions and deletions in equal proportions (left panels), when compared to alignments where the gaps result from largely deletion events (right panels). This is evident by noting that the dots of a given color (G/S value) are higher in the charts in the left panel and lower in the right, for any given pair of inference methods being compared. However, there are distinct differences among the methods too, and they are brought out in these pairwise comparisons. For instance, it is clear that, irrespective of whether the insertion-deletion ratio is 1:1 or 1:3, in general, the Bayesian, MP and PhyML methods are somewhat comparable, while NJ does the poorest in the presence of gaps, especially when G/S is large. However, comparing the relative performance of the methods from such graphs becomes subjective. Therefore, we conducted the paired *t*-test (at 5% level of significance) for each of the 110 data points (parameter combinations or "genes") in each of the graphs. The results of the *t*-test are given by means of a letter that signifies if a particular method is statistically better than the other in a given comparison (B – Bayesian analysis, P – PhyML, L – ML*ε*, M – Maximum Parsimony, and J – Neighbor-Joining). We also determined which method was better, overall, in each of the panels, using the Z test, and this is shown by the corresponding letter with an asterisk in the upper triangle.

Thus, in the comparison between NJ and PhyML, we see that PhyML shows a significantly greater overall accuracy (Z test; *p *< 0.001), and that this difference is almost always statistically significant for the individual comparisons. The superiority of PhyML over NJ in the presence of gaps is very clear when the insertion-deletion ratio is 1:1 (all 110 comparisons statistically significant; *t *test; *p *< 0.05). When the ratio is 1:3, again, PhyML is better than NJ almost all the time; NJ is found to be significantly better only in two instances out of 110; two comparisons were not significant. The comparison between MP and NJ also yields similar results, with MP being clearly superior most of the time (for 78 "genes", with 32 comparisons turning out non significant; NJ is never better than MP.) in the left panel. The result in favor of MP is more pronounced at higher G/S values, with the graph deviating away from the diagonal. In the comparison between MP and PhyML, PhyML is superior to the other in a majority of cases, irrespective of the insertion-deletion ratio. Interestingly, MP superiority is seen only at very high G/S values, while PhyML is better almost everywhere else. This is particularly evident in the right panel (insertion-deletion rate ratio of 1:3). In both panels, PhyML is significantly better, overall (Z test; *p *< 0.001).

**Figure 5:**
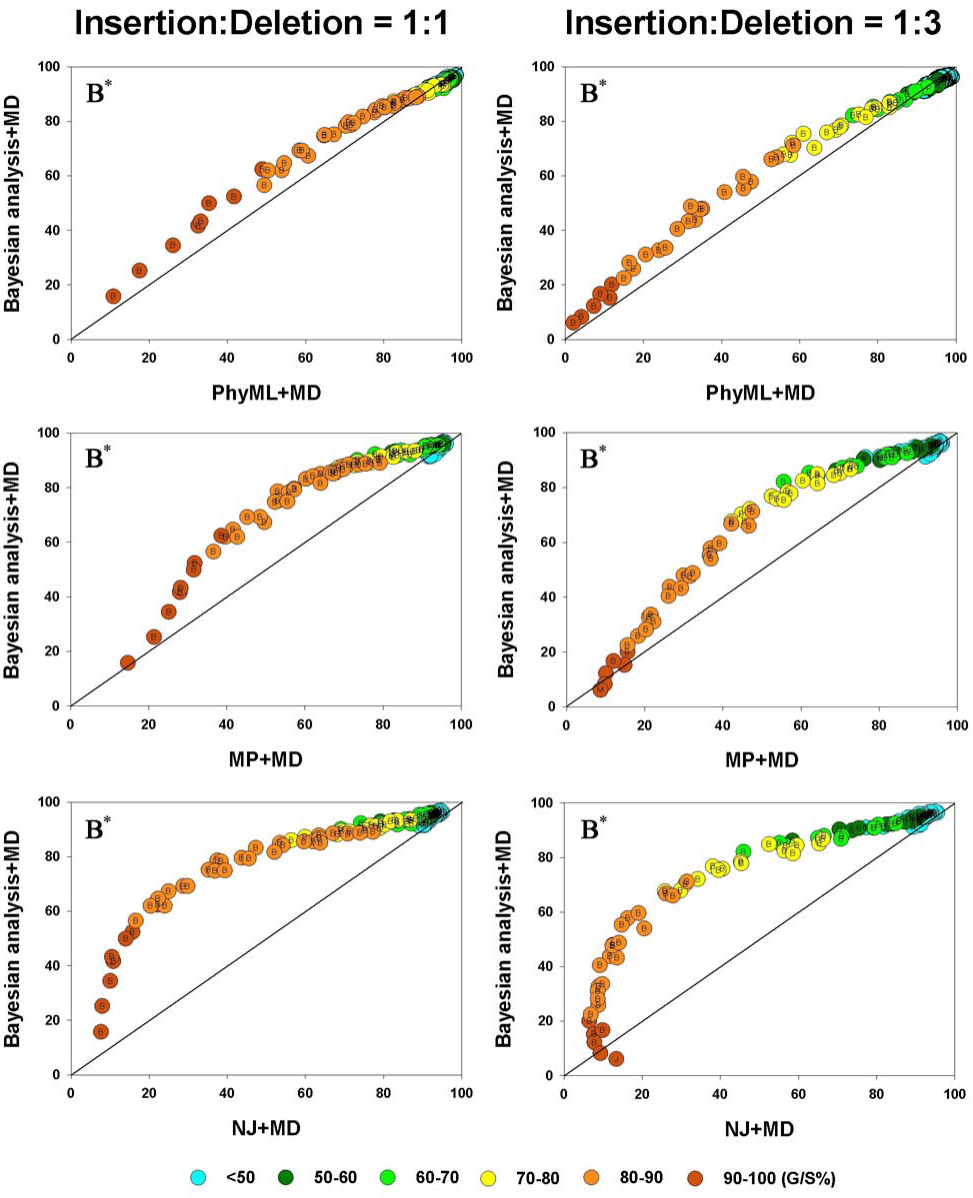
**Pairwise comparison of inference methods under MD gap treatment**.  values are compared, in a pairwise fashion, for four inference methods: Bayesian analysis, NJ, PhyML, and MP. As elsewhere, the left and right columns refer to the 1:1 and 1:3 insertion-deletion rate ratios. In each panel, the average phylogenetic accuracy, , for one inference method is plotted against that of another. The dots in each graph are color coded to reflect the gap percentage (G/S %) against which the  values have been measured, ranging from light blue (for the lowest G/S values) to red (for the highest G/S values); see legend below figure. Each  value reflects one of all possible combinations of values of substitution rate, *r *and indel rate, *λ*, (see Additional file 1), sequence length, *l *= 500, transition-transversion rate ratio, *κ *= 2, and the gamma among-site rate variation shape parameter, *α *= 0.5, averaged over 100 replicates, for a total of 110 data points in each graph. The paired *t*-test (*p *< 0.05) results are shown with a letter (within the dot) that signifies if a particular method is statistically better than the other in a given comparison (B – Bayesian analysis, J – Neighbor-Joining, P – PhyML, and M – Maximum Parsimony) for the parameter combination. The *t*-test results that were not statistically significant are presented with no symbol (letter) within each dot. The results of the Z test (*p *< 0.001) over 110 data points for each method-method comparison is shown with a letter followed by an asterisk (B* – Bayesian analysis, J* – Neighbor-Joining, P* – PhyML, and M* – Maximum Parsimony)

In Figure 5, we show the results of the Bayesian method under the MD treatment compared to PhyML, MP, and NJ methods. The figure shows that, irrespective of the insertion-deletion rate ratio and the G/S value, the Bayesian method is more accurate than MP, NJ or PhyML, overall (*p *< 0.001). When compared individually, it is seen to be better than NJ in all the genes in the left panel and all but one of the genes in the right panel. Next, the Bayesian method is seen to be better than PhyML, overall, but it is statistically better (*p *< 0.05) 48 times (out of 110 comparisons across the entire spectrum of G/S values), with PhyML outperforming it (*t *test; *p *< 0.05) in 22 cases, with neither being better than the other in the remaining 40 genes, in the right panel. In the left panel (1:1) similar results were obtained, where PhyML and Bayesian were each statistically better (*t *test; *p *< 0.05) than other roughly equal number of times (around 30 cases each), with the two methods occupying different "niches" (Bayesian doing better at high gap percentages and PhyML at the low to intermediate levels of gap percentage.). When Bayesian and MP methods are compared, the Bayesian analysis is statistically better (*t *test; *p *< 0.05) in almost 90 cases, whereas MP is better almost never, irrespective of the insertion-deletion rate ratio.

It is important to note that the MD treatment is different among the inference methods. (In the case of the probabilistic methods – PhyML and Bayesian analysis, the likelihood is summed over all four nucleotides at the gapped sites [27-29], while for a distance method like NJ, the nucleotide state at each gap is inferred by distributing the missing changes to unambiguous changes, and finally, for the MP method, a given state is assigned to each gapped site in a sequence if it is the most parsimonious, given the placement of the taxon in the tree (see the FAQ on the PAUP*website . Hence, it appears that the level of accuracy seen in this study for the different inference methods is also attributable to the accuracy with which the state is inferred/likelihood is computed at the gaps by the corresponding MD methods.

**Figure 6:**
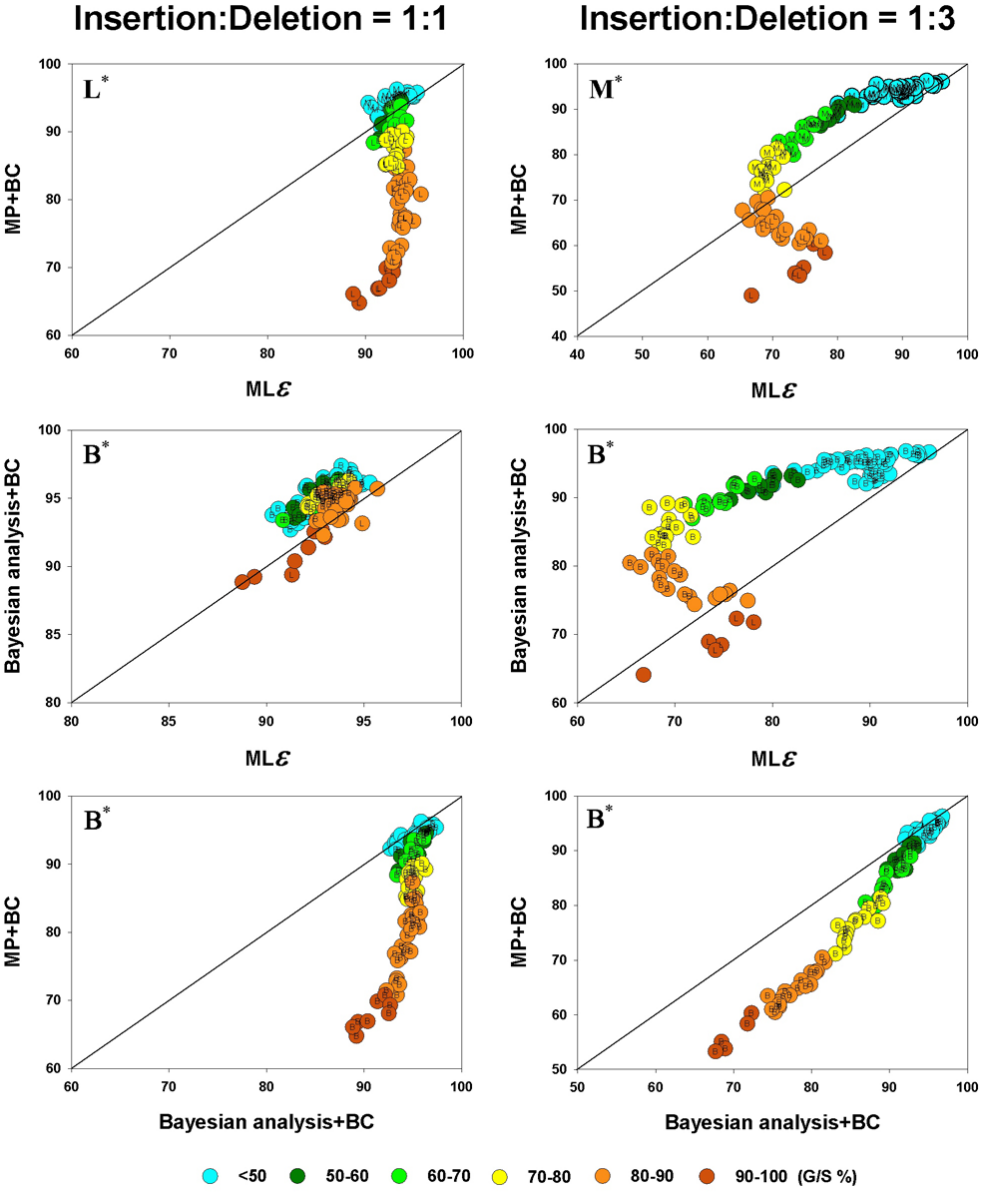
**Pairwise comparison of inference methods when gapsare coded as distinct evolutionary events**.  values are compared, in a pairwise fashion, for the inference methods: MP, Bayesian analysis, and the ML*ε *analysis, when the gaps were treated as binary characters or by the DNAML*ε *method. As in Figure 4 and 5, the average phylogenetic accuracy, , for one method is plotted against that of another in each panel. For each pairwise comparison between the inference methods, the left panel shows the results for the insertion-deletion rate ratio is 1:1 and the right panel when it is 1:3. The dots in each graph are color coded to reflect the gap percentage (G/S) against which the  values have been measured, ranging from light blue (for the lowest G/S values) to red (for the highest G/S values). Each  value reflects one of all possible combinations of values of substitution rate, *r *and indel rate, *λ*, (see Additional file 1), sequence length, *l *= 500, transition-transversion rate ratio, *κ *= 2, and the gamma among-site rate variation shape parameter, *α *= 0.5, averaged over 100 replicates, for a total of 110 data points in each graph. The paired *t*-test (*p *< 0.05) results are shown with a letter (within the dot) that signifies if a particular method is statistically better than the other in a given comparison (B – Bayesian analysis, L -ML*ε*, and M – Maximum Parsimony) for the parameter combination. The paired *t*-test results that were not significant are presented as dot with no symbol. The results of the Z test (*p *< 0.001) over 110 data points for each method-method comparison is shown with a letter followed by an asterisk (B* – Bayesian analysis, L* – ML*ε*, and M* – Maximum Parsimony).

Next, we compare the accuracy of the different inference methods, again, taken two at a time, when gaps are treated not merely as missing data but as information that is included in the phylogenetic analysis. Under the MP and Bayesian methods, gaps can be treated as binary characters (BC), with sites in a given column being scored as a 1 if gapped and 0 if not. Among the recent advances in the modeling of molecular sequence evolution is the integration of insertion and deletion events along with base substitution processes in a probabilistic framework for phylogenetic inference [23]. We have used this method for maximum likelihood analysis of our data, to compare this treatment (which we refer to as ML*ε*) with the BC treatment in MP and Bayesian analyses. These comparisons (again, pairwise as in Figure 4 and 5) are shown in Figure 6.

As in Figure 4 and 5, here too the average phylogenetic accuracy, , for one method is plotted against that of another in each panel (1:1 and 1:3), and the color-coding scheme is the same as well. Figure 6 shows that the differences among the inference methods in accuracy in the presence of indel-induced gaps, is much more evident when gaps are included in the phylogenetic analysis and not treated as missing data (that is, when compared to the results in Figure 4 and 5). Furthermore, the accuracy is generally much higher, even in the method with the lower accuracy (note the  value ranges in the axes, which have been optimized for maximum spread of the points within each graph for greater visibility). Finally, it can be seen, just as in Figure 4 and 5, but in a more pronounced manner, that the accuracy of both methods is higher for a given G/S level when the gaps result from equal proportion of insertions and deletions (panels in left column), as opposed to when they are largely from deletion events (right column), as evidenced by a comparison of the heights of the dots of a given color between the two panels, particularly at the mid to higher G/S values (light green, yellow and orange colored dots).

The different indel-coding methods are compared for their performance, in conjunction with the corresponding inference methods, in Figure 6. It is immediately obvious that accuracy is much higher (and in a tight range of values) in all the left panels (1:1 ratio) for the probabilistic methods (Bayesian and ML*ε*) when compared to the right side panels (1:3 ratio), providing a compelling case for the association between phylogenetic accuracy and evolutionary origin of alignment gaps (insertion or deletion) – at least for the probabilistic methods. This fact is even more obvious in the middle left panel where the two probabilistic methods are compared. When MP and ML*ε *are compared (Figure 6, top panels), it is clear that ML*ε *has a much higher accuracy in the medium to high range of G/S values when the insertion-deletion ratio is 1:1, with MP doing better at low to medium G/S values. This difference between the two methods is much more pronounced in the right panel (insertion-deletion ratio, 1:3), where ML*ε *is better only at the highest G/S values and MP clearly the better of the two elsewhere. The middle panels show that Bayesian analysis produced more accurate trees when compared to ML*ε *in 77 (right panel) and 96 (right panel) out of 110 comparisons. Just as in the MP, ML*ε *comparison (top panels), ML*ε *again outperforms Bayesian at the highest G/S values. The difference between the two panels is quite evident, with both methods varying in accuracy in a very tight range in the left panel (when compared to the 1:1 ratio). Note that the distribution of  values is somewhat similar when the MP and Bayesian methods are each compared to ML*ε *(top and middle panels for the 1:3 ratio), suggesting a similar pattern between MP and Bayesian, under BC treatment of gaps, although the Bayesian method appears to be doing better than MP against ML*ε*. These two methods are compared in the bottom panels. As mentioned above, it is immediately apparent that the accuracy for the Bayesian method in the left panel is much higher (minimum 90%) when compared to the right panel. These panels also show that whenever the difference in accuracy between MP and the Bayesian method is statistically significant (*t *test; *p *< 0.05), the latter is always better (with 30 percent of the cases being non significant). Furthermore, the "genes" where the difference in accuracy is statistically significant are mostly spread across medium to high G/S values. In summary, the Bayesian method is superior to the MP and ML*ε *methods under the gap coding approach, irrespective of the relative proportions of insertions and deletions in the alignment.

The results shown in Figures 4, 5, and 6 have been obtained from our analyses of the alignments obtained from simulations done on the balanced (symmetric) model tree (Figure 1). We also obtained sequence alignments from simulations done with 16-taxon random-branching and pectinate trees for a subset of parameter values that, however, spanned the range of parameter values used in this study (see Additional File 1). All the analyses shown in Figures 4, 5, and 6 were done on these alignments as well (not shown), including the pairwise comparisons among the inference methods and the paired *t*-tests. The results in those analyses showed that while the inference methods compare among themselves for the random-branching tree just as they did for the balanced tree, there are some differences in the case of the pectinate tree. In the case of the MD analysis, while Bayesian was the better method overall, the performance of PhyML in the case of the pectinate tree was glaringly different. In the case of the balanced tree, PhyML showed greater accuracy than NJ in essentially all the cases, irrespective of the insertion-deletion ratio. However, for the pectinate tree, the roles are exactly reversed, with NJ better than PhyML in essentially all the cases – again, irrespective of the insertion-deletion ratio. Similarly, while PhyML and MP were each better than the other roughly equal number of times in the case of the balanced tree, MP accuracy was superior for the pectinate tree in essentially all the genes studied, irrespective of the insertion-deletion rate ratio.

When indel coding was used, again, the random tree results are quite similar to those from the balanced tree. Interestingly, the results from the pectinate tree were not glaringly different from those from the balanced tree, but rather, the two were largely similar, except that the overall accuracy was lower by about 20 percent.

## Discussion

We undertook this study to investigate the relationship between the number of gapped sites in a sequence alignment and the accuracy of phylogenetic inference, and furthermore, to understand the impact of different gap treatment methods, phylogenetic inference methods, the ratio of insertions to deletion events in the evolutionary history of the sequences, and other sequence parameters such as sequence length and the transition-transversion rate ratio, on this relationship. Using the computer program, Dawg version 1.2 [30] we simulated DNA evolution along a 16-taxon model tree (Figure 1), incorporating both nucleotide substitution events and insertion and deletion (indel) events (the latter as a function of the substitution rate.). The resulting DNA alignments were then subjected to three gap treatment methods, namely, MD, BC, and ML*ε*, and the phylogenetic analysis was done using popular phylogenetic inference methods – distance (NJ), parsimony (MP), likelihood (PhyML) and Bayesian analysis.

A remarkable result in this study is the strong, almost deterministic, dependence of the accuracy of phylogenetic inference on the percentage of gapped sites in the alignment, irrespective of the inference method, gap treatments, or insertion-deletion rate ratio, when the percentage of gapped sites was high (Figure 3). This made the assignment of gap thresholds for specific levels of phylogenetic accuracy fairly straightforward, without being necessarily concerned with other determinants of phylogenetic accuracy. It was only at lower gap levels that the relationship was not as straightforward, and other factors (e.g., substitution rate) began to play a part in directly influencing the accuracy of the inferred trees (as evidenced by the contour lines of accuracy crossing gap percentage thresholds in Figure 3).

Earlier studies that have compared gap treatment methods have been confined to comparing their relative performances within a given inference method, particularly MP [5,6]. Therefore, this study was undertaken to provide users with a comparison of other commonly used inference methods as well. We find that the probabilistic methods are clearly superior to MP and NJ, irrespective of whether gaps are treated as missing data or binary characters. Treating gaps as binary characters implies the assignment of unambiguous phylogenetic signal to them in the evolutionary history of the sequences. Therefore, the number of gaps has little bearing on the distortion of the phylogenetic signal under the BC method. On the other hand, the MD method requires the inference of the missing state at each gapped site (or the summation of the likelihood for all four nucleotides at the gapped sites), a process that is bound to be strained with increasing number of gaps in the alignment. Therefore, it is easy to understand the relative superiority of the BC gap treatment method. It must be noted, of course, that this method can only contribute to phylogenetic accuracy as long as the alignment gaps are known without error (as in this study). Thus, the importance of the accuracy of sequence alignment cannot be underestimated.

The ML*ε *method performed well in our study, although the Bayesian method was better, especially when the insertion-deletion ratio was 1:3 (Figure 6). When compared to MP analysis (the other inference method that incorporated the BC), ML*ε *was much better when the number of gaps was high, irrespective of the insertion-deletion ratio. Such methods hold the potential for more accurate reconstruction of phylogenies in the presence of large alignment gaps (also see [15,31]).

In addition to the MD treatment and the gap-coding treatments such as BC, other treatment methods exist, although not widely used anymore. One of these is pairwise deletion, a gap treatment method that is meaningful only when sequences are compared in a pairwise fashion, as in distance methods of inference, such as NJ. Moreover, it is an extremely rapid method that is suited to the speed of NJ. The other is complete deletion of entire columns of gapped sites from the alignment, which is a gap-treatment method that is applicable to any phylogenetic inference method. We did these analyses as well, because there is sometimes an uncertainty about which of these two methods is better [2,32]. The complete deletion of gaps posed a problem in our study as the number of sites that needed to be removed from the alignment, especially at higher substitution rates, caused the remaining sequence length to become so small that often at least one of the four nucleotides failed to be represented in the alignment. Therefore, we used this method only when the substitution rate was very low (*r *≤ 0.2), and when the alignment length (remaining after complete deletion) for each replicate of a given sequence combination was at least 100 nts.

Since the complete deletion treatment could be used only for low substitution rates, the comparison between the two treatments is also made only across this range. Furthermore, since the pairwise deletion method can only be used in conjunction with the NJ method in this study, we compared the two methods only for NJ. Both methods are comparable at low to moderate gap percentages, but diverge thereafter in the accuracy of phylogenetic inference (not shown). It must also be noted that the gap percentage does not reach very high levels in the pairwise deletion as it does in the complete deletion method. Thus, while for a given gap percentage, the two treatment methods may be comparable in terms of phylogenetic accuracy, the pairwise removal of gaps appears to be better since the gap percentage is much lower with this method.

A comprehensive list and analysis of gap treatment methods may be found in Ogden and Rosenberg [6] and Simmons Muller and Norton [5]. However, they did not compare among phylogenetic inference methods, even for those gap-treatment methods that were common to multiple inference methods. In this study, while we do compare among gap-treatment methods, our emphasis is also on comparing among inference methods, insertion-deletion ratios, and the effect of the amount of gap on phylogenetic accuracy under varying parameters.

In order to better understand the influence of the alignment gaps on phylogenetic accuracy, we performed the same simulations, but with only base substitutions and no indels. As there were no gaps in the alignments, the data were subjected to phylogenetic analysis without any processing by means of gap treatment methods. The results of this analysis showed that, as expected, Maximum Likelihood and Bayesian analysis produced the most accurate trees, particularly at the highest substitution rates (not shown).

Another notable finding in this study is the differential influence of insertions and deletions on phylogenetic accuracy. Most of the commonly used gap treatment methods do not distinguish between insertions and deletions. Our results show that phylogenetic accuracy was lower when the insertion-deletion ratio was 1:3. Even the probabilistic methods (PhyML, ML*ε *and Bayesian), which produced the most accurate trees when insertions and deletions were introduced in equal numbers, performed somewhat poorly when the ratio was 1:3 (Figure 3, 4, 5, 6). It therefore, appears important to develop methods that first distinguish between insertion and deletion events in the evolutionary history of the sequences in an alignment, and then treat them separately to add distinct signals to the phylogenetic analysis.

**Figure 7:**
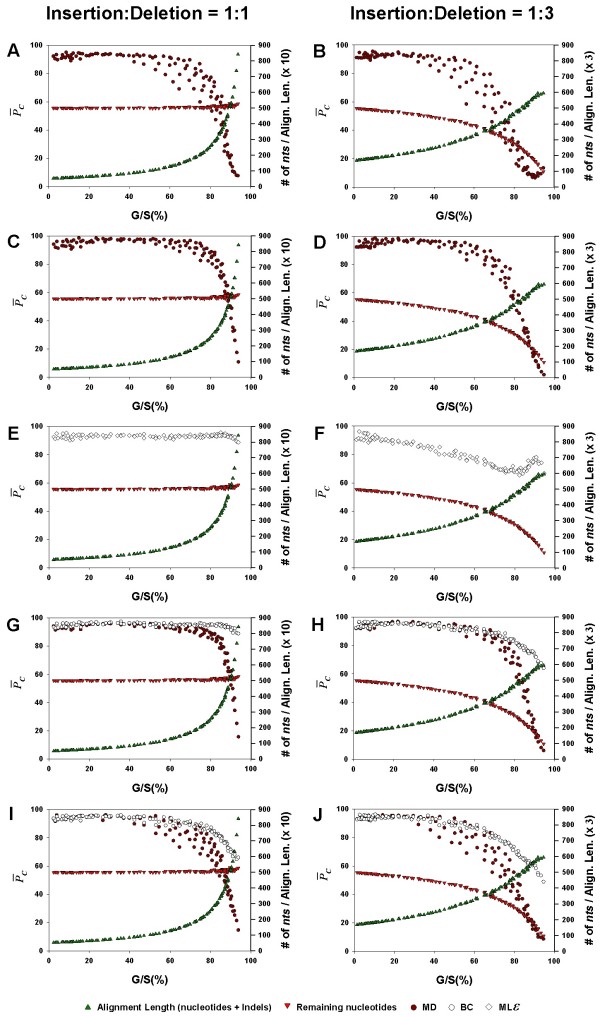
**Effect of the alignment gap percentage on phylogenetic accuracy, number of characters remaining in the alignment, and total alignment length after gap-introduction**. The average accuracy, , (dark red circles for the MD treatment, open diamonds for the ML*ε *method, and open circles for the BC treatment), remaining number of characters in the alignment after the gaps are removed (red inverted triangles), and the total length of the alignment (including gaps; symbolized by green, upright triangles), are each shown as a separate function of the average gap percentage, for NJ (Panels A, B), PhyML (C, D), ML*ε *(E, F), Bayesian (G, H), and MP (I, J) inference methods. The left panels show the results for the insertion-deletion rate ratio of 1:1 and the right panels for the ratio 1:3. Each data point in the graph was obtained as the corresponding value for one of all possible combinations of values of *r*, the substitution rate and *λ*, the indel rate (see Additional file 1), sequence length (*l *= 500), transition-transversion rate ratio (*κ *= 2), and the gamma among-site rate variation shape parameter (*α *= 0.5), averaged over 100 replicates, for a total of 110 data points in each graph.

In this study, the metric we have used to measure the accuracy against is the percentage of gaps in the alignment, and this in turn has been measured mainly as G/S. Some studies have found that it is not the amount of data missing but rather the amount of data remaining that matters in determining the accuracy of the phylogeny being inferred [10,12,33]. In order to compare our results with the results from these studies, we show the accuracy, , the remaining number of nucleotides after the gaps are removed from the alignment, and the total length of the alignment resulting from the introduction of indels during the evolution of the sequences, all plotted as a function of G/S (Figure 7). The layout of Figure 7 is the same as that of Figure 3, with the left and right columns referring to insertion-deletion ratios of 1:1 and 1:3, respectively, and the inference methods arranged one below the other, in the same order, namely, NJ, PhyML, ML*ε*, Bayesian analysis and MP.

One of the first things that stand out in Figure 7 is the general accuracy of the MD method when the G/S is low and poor accuracy when G/S is high, irrespective of the inference method. Interestingly, when the accuracy curve in each graph is compared to the curve of the remaining number of nucleotides, there seems to be little relationship between the two in the left panels (1:1), again, irrespective of the inference method. Thus, even as the number of remaining nucleotides (red triangles) continues to be high for large G/S values, the  value (for MD treatment; dark red circles) plummets down to close to zero. This is because, although the remaining number of nucleotides is high, this is largely a consequence of insertion events having added nucleotides to the sequences. Thus, although there is data, there is little phylogenetic information in it, since homology across sequences at these levels becomes nebulous, at best, leading to low accuracy. On the other hand, the curve of remaining nucleotides itself drops with increase in the G/S value in the right column panels (1:3) – a reflection of the greater proportion of deletion events. Therefore, while the  values drop with increase in G/S in spite of an abundance of data in the left column panels, they do so in the right column panels evidently because of the loss of data as G/S increases (note the scale on the secondary Y axis). Thus, while the remaining amount of data may be an important determinant of accuracy (as in the right column in Figure 7, and as mentioned in [10,12,33]), this is true only when homology among the sequences in the alignment can be established in the remaining character data. If, however, the remaining character data is largely a result of insertion events, the relationship is unlikely to hold, as seen in the left panels.

On the other hand, if the gaps are coded separately (e.g., as BC), then the phylogenetic signal present in the gaps (if the alignment is accurate) increasingly becomes the only information for the inference method to rely on, as G/S increases. The loss of signal from the character data is reflected in the decreased phylogenetic accuracy at high G/S values (left column). The greater loss of phylogenetic accuracy at medium G/S values in the right column panels of Figure 7 can be attributed to fewer deletion events that are distinct and non-overlapping when compared to insertion events that are more likely to be distinct and non-overlapping, as the increase in the total length of the alignment with indel introduction will be much higher when the insertion-deletion rate ratio is 1:1.

In this study, we also found that the alignments from the random-branching tree yielded essentially the same results as those from the balanced tree, while those from the pectinate tree were different (not shown). The analyses from the pectinate tree data in general showed lower accuracy than the corresponding analyses from the balanced tree datasets. Furthermore, the relative performances of the different inference methods were not the same between the two model topologies. In particular, the relative performance of the PhyML method was worse when the topology contained pectinate branching.

This is a simulation-based study and is confined to certain specific simulation parameters and methods of gap treatment and phylogenetic inference used in this study. However, the choices of the parameter values have been made based on empirical studies in the literature. This included the size distribution of indels as well [18], which may not be a critical feature as far as the BC treatment is concerned, but may be important when the state is inferred at the gaps or coded. Therefore, we believe that the results obtained in this study are sufficiently general to be useful to the community of molecular phylogeneticists. However, we must add a note of caution that while it is likely that the general results of this study will hold, the particulars may be dependent on the specific choices of simulation and other parameter values. Finally, the relationships between phylogenetic accuracy and gap percentage in this study were derived based on two unlikely events in empirical studies – knowledge of the true tree and a perfect alignment. These certainly are sources of uncertainty and/or error in real data analysis, and must be accounted for, in empirical studies. However, the utility of simulation-based studies such as this is that they serve to provide an assessment and quantification of relationships in the absence of confounding factors.

## Conclusion

The presence of gaps in molecular sequence alignments is common-place in the literature. Our simulation-based results show that, when the alignment gaps reflect indel events without error, and the number of gapped sites per sequence is ≤20 percent of the sequence length, all the inference methods used (NJ, PhyML, ML*ε*, Bayesian analysis and MP) perform well in accurately inferring the phylogeny. However, when the number of gaps is large (≥80 percent), the Bayesian method clearly outperforms the other inference methods when the gaps are treated as Missing Data (MD), although it must be noted that since each inference method uses a different criterion in treating gaps as missing data, the higher accuracy for the Bayesian and PhyML method can perhaps also be attributed to a more accurate integration of the state at each of the gapped sites. Within the MP and Bayesian methods, the inference of the phylogeny was significantly more accurate when each gapped site was treated as a Binary Character state (BC) than when the gaps were treated as MD. When the sequences in an alignment contain a large number of gaps, as in the case of highly diverged sequences, coding gaps as in likelihood analysis (ML*ε*) may be more efficient than Bayesian or MP in combination with the BC method. Finally, our results also show that it is more difficult to accurately infer the phylogeny from an alignment where a greater proportion of gaps reflect deletion events rather than insertion events in the evolutionary history of the sequences in the alignment.

## Methods

### Computer simulations

True DNA sequence alignments were generated by simulating evolution along a 16-taxon model tree using the computer program, Dawg, version 1.2 [30]). The model tree topology used for the simulations was a balanced, non-ultrametric tree with random branch lengths (Figure 1), borrowed from [34]. The nucleotide substitution model used was HKY [35], with rate heterogeneity among sites. Simulations were done to mimic nucleotide sequences with different properties by systematically varying the sequence and indel parameters (in a fully factorial manner, see below). Simulations were also done with 16-taxon random-branching and pectinate trees, and these alignments were also subjected to the same analyses that the alignments from the balanced tree of Figure 1 were. The results of these analyses showed that while the alignments from the random-branching tree were essentially the same as that from balanced tree, those from the pectinate tree were not. These differences have been pointed out at appropriate parts in the text, while presenting the results only from the balanced tree of Figure 1.

The values of the sequence and indel parameters used in the simulations are given in Additional file 1. These values were varied based on several studies (e.g., [18,20,36,37]) to ensure the generality of the conclusions from this study. The sequence parameters varied were: initial sequence length (*l*), transition to transversion rate ratio (*α*), and the rate of nucleotide substitution (*r*) as the number of substitutions per site. The shape parameter (*a*) of the gamma distribution was set to 0.5 to specify the extent of rate heterogeneity among sites. The nucleotide base frequencies were kept constant (A% = T% = 30%; G% = C% = 20%) throughout the simulations, which were based on the literature [18,19]. All other options in the program pertaining to the sequences were set to default during simulation. Note also that the results, when compared between *l *= 500 and *l *= 2500, produced similar patterns (except for an increase in accuracy), as also when compared between *κ *= 2.0 and *κ *= 5.0. Therefore, results have been presented in this paper for only *l *= 500 and *κ *= 2.0.

The rate at which indels were introduced during simulation, *λ*, was varied as a function of the substitution rate (see Additional file 1). For example, a *λ *of 0.03 refers to an average of 3 indels per 100 substitutions. *λ *was also varied to include the range typically observed in empirical sequence data [17-20]. In addition, in order to mimic the very large number of gaps that can potentially be seen in introns and other non-coding sequences, a few higher indel rates were also added (with corresponding increased substitution rates). Although phylogenetic analysis is typically done without differentiating between insertions and deletions, the insertion to deletion ratio was set to either 1:3 [18,20] or 1:1 [21,22,38], at a given indel rate, in order to accommodate differing opinions about the ratio of deletions and insertions, and to determine if the two have different impacts on phylogenetic accuracy. The size distribution of insertions/deletions was as per mammalian pseudogene data [18] and ranged from 1 to 60 bp in length. This distribution of the indel length can be observed in other non-coding sequences, such as chloroplast inter-genic regions [19], and nuclear DNA sequences of primates[22]. Each set of sequence and indel parameters (44 sets and 20 sets, respectively) was replicated 100 times, thus producing 88,000 16-taxon non-coding sequence alignments.

### Phylogenetic analysis

Phylogenetic analysis was done on the alignments obtained using Neighbor-Joining (NJ) and Maximum Parsimony (MP) methods as implemented in PAUP* version 4.0 b10 [4]. Maximum Likelihood analyses (PhyML) was done using the program, PhyML version 2.4.4 [27], because of its speed [27]. Finally, Bayesian analysis was done using MrBayes version 3.1.2 [28,29,39] with default settings.

Maximum Likelihood HKY pair-wise distances were used for the NJ analyses. In PhyML analysis, the initial tree was built using BIONJ [27]. The parameters of the HKY substitution model (the four base frequencies and the transition/transversion rate ratio) along with the proportion of invariable sites and the gamma distribution shape parameter were estimated from the simulated data for both NJ and PhyML analysis. For the MP analysis, a heuristic search was done using the stepwise addition algorithm for the provisional tree and subsequent branch swapping using the Nearest-Neighbor Interchange (NNI) method. (NNI results for MP are known to be as good as those from the more thorough – and time-consuming – Tree Bisection Reconnection (TBR) searches [40,41]. In addition, our TBR and NNI results for a representative subset of the simulations yielded essentially the same results). All other settings were set to default. Similarly, results analyzed from the PhyML version 2.4.4 [27] using NNI were not different from the recent PhyML version 3.0 [27] with SPR (Subtree Pruning and Regrafting) tree search.

For the Bayesian analysis, the nucleotide substitution model used was HKY with invariant sites and rate heterogeneity of rates across sites. The number of generations was set to 50,000 with a sampling frequency of 50. In cases when convergence was not obtained (typically for high substitution and indel rates), the number of generations was increased to 100,000 with a sampling frequency of 100. Burn-in was set to 25 percent of the generations and the inferred tree was estimated as the consensus of all compatible groups of the post burn-in trees. The inferred tree was then compared to the model tree and topological distances were measured using PAUP* version 4.0b10 [4].

### Treatment of gaps

Gapped sites in our (true) alignments were subjected to the following gap treatment methods during phylogenetic analysis: a) "MD" (Missing Data) – in this treatment the nucleotide state at each gapped site is treated as a missing character based on the optimization criteria, based on whether the inference method is distance-based, parsimony, likelihood, or Bayesian [4,27,28]. Treating gaps as unknown or missing data is the default option in PAUP* The FAQ page for PAUP* at  explains the working of this treatment under each inference method, and for PhyML and Bayesian, it is explained in [27-29]. Briefly, PAUP* deals with missing characters in the following manner: under the parsimony criterion, a missing character in a sequence is assigned the most parsimonious state given its placement in the tree. Under the likelihood criterion, a gapped site is assigned a state based on the likelihood which is computed by summing the likelihoods over all possible states – a strategy that is used by PhyML as well. For distance methods, PAUP* deals with the missing data by distributing the missing or ambiguous changes proportionally to each unambiguous change. Bayesian analysis was done using the program MrBayes [28,29,39], which deals with gaps just as other Maximum Likelihood programs [4,42-44]. (b) "BC" (Binary Character state) – the gapped sites in each column are coded as binary characters (1 if gap present, 0 if absent), available for the MP and Bayesian methods [4,28,29]. This treatment can be invoked in PAUP* for MP analysis with the commands "GapMode = Missing", and "Symbol = 01" under "Format" and "Options", and providing a matrix of symbols reflecting gapped sites. In Bayesian analyses (using MrBayes), binary characters are included as a separate binary restriction data partition, using the command "coding = variable" under "lset". (c) "ML*ε *", a probabilistic model implemented in the DNAML package [23] of PHYLIP [44] program, that incorporates insertion and deletion events in addition to substitution events in the evolutionary model.

### Assessing phylogenetic accuracy

The accuracy of the inferred trees was measured as the percentage of internal branches reconstructed correctly in the inferred tree, obtained as , where *d*_T _is the topological distance between the inferred and model trees [45,46] and *m *is the number of sequences in the alignment (16). *P*_C _values were averaged over all the (100) replicates for each parameter combination, to give . In MP analysis, when multiple equally parsimonious trees were recovered, all comparisons were made between the model tree and a randomly chosen single tree from among equally parsimonious inferred trees, since the strict consensus of these trees tended to produce a star tree, especially at high substitution rates [10].

## Authors' contributions

SRG conceived and designed the study. BD did the simulations, conducted the analyses, and wrote the first draft of the manuscript. Both authors read and approved the final manuscript.

## Supplementary Material

Additional file 1 **Sequence and indel parameter values used in the generation of gapped sequence alignments by computer simulation**. The sequence length, *l*, is measured as the number of nucleotides. The indel rate, *λ*, refers to the number of indel events per nucleotide substitution, and is expressed as a proportion (for example, a *λ *value of 0.03 indicates that there were three indel events for every 100 substitutions), *r *is a multiplier, that, when multiplied by a given branch length in the model tree and the sequence length, yields the number of substitutions to be introduced in that branch during simulation. Click here for file
