# Supplementary material for: Phylogenetic inference under varying proportions of indel-induced alignment gaps
Source: BMC Evol Biol. 2009 Aug 23;9:211. doi: 10.1186/1471-2148-9-211 (PMC2746219; doi:10.1186/1471-2148-9-211)
Supplement: Additional file 1 — Sequence and indel parameter values used in the generation of gapped sequence alignments by computer simulation. The sequence length, l, is measured as the number of nucleotides. The indel rate, λ, refers to the number of indel events per nucleotide substitution, and is expressed as a proportion (for example, a λ value of 0.03 indicates that there were three indel events for every 100 substitutions), r is a multiplier, that, when multiplied by a given branch length in the model tree and the sequence length, yields the number of substitutions to be introduced in that branch during simulation. [file 1471-2148-9-211-S1.pdf]

# Additional files

**Additional file 1.pdf**: Sequence and indel parameter values used in the generation of gapped sequence alignments by computer simulation. The sequence length, *l*, is measured as the number of nucleotides. The indel rate, λ, refers to the number of indel events per nucleotide substitution, and is expressed as a proportion (for example, a λ value of 0.03 indicates that there were three indel events for every 100 substitutions). *r* is a multiplier, that, when multiplied by a given branch length in the model tree and the sequence length, yields the number of substitutions to be introduced in that branch during simulation. File is formatted as word document (.doc)

**Additional file 1. Sequence and indel parameter values used in the generation of gapped sequence alignments by computer simulation**

| Sequence Parameters | Values |
|---|---|
| Initial sequence length ($l$): | 500, 2500 |
| Transition-transversion rate ratio ($\kappa$): | 2, 5 |
| Gamma distr. shape parameter ($\alpha$): | 0.5 |
| Nucleotide subst. rate multiplier ($r$): | 0.025, 0.05, 0.1, 0.2, 0.4, 0.8, 1.0, 1.25, 1.5, 1.75, 2.0 |
| **Indel Parameters** | **Values** |
| Indel rate ($\lambda$): | 0.03, 0.05, 0.07, 0.09, 0.11, 0.13, 0.15, 0.17, 0.19, 0.25 |
| Insertion:Deletion rate ratio: | 1:1, 1:3 |
